# Learning Surgical Skills Through Video-Based Education: A Systematic Review

**DOI:** 10.1177/15533506221120146

**Published:** 2022-08-14

**Authors:** Samy Cheikh Youssef, Abdullatif Aydin, Alexander Canning, Nawal Khan, Kamran Ahmed, Prokar Dasgupta

**Affiliations:** 1 Guy’s, King’s and St Thomas’ School of Medical Education, King’s College London, London, UK; 2 Guy’s Hospital, King’s College London, 215816 MRC Centre for Transplantation, London, UK; 3 Department of Urology, 40470 The London Clinic, London, UK

**Keywords:** surgical education, surgical skills, video based surgical education, surgical video, surgery

## Abstract

**Background:**

Educational videos are a potent resource for the learning of surgical skills among different study cohorts. However, there is limited evidence on the effectiveness of different educational video interventions and their features.

**Methods:**

A systematic search of MEDLINE (via PubMed), Embase (via OVID), Cochrane libraries and Clinicaltrials.gov was performed from inception to 28/02/21. Studies included were not limited by date of publication, studies aiming to assess the impact of video-based interventions in the direct acquisition of surgical skill were included. Eligible studies were analysed based on study type, type of video intervention, method of assessment and period of education. The educational impact of the studies was also assessed as per Messick’s framework for testing validity of evaluation methods and McGhagie’s model for analysing translational outcomes.

**Results:**

22 studies were deemed suitable for inclusion, of which 14/22 (63.6%) demonstrated a significant improvement in knowledge/skills following the video-based teaching interventions, 3/22 (13.6%) studies demonstrated an improvement in trainee satisfaction scores. A recurrent limitation of the included studies was the lack of validation of selected assessment methods. None of the included studies scored on all 5 parameters of validity as defined by Messicks validity framework. Furthermore, none of the included trials were conducted for long enough to indicate direct changes to patient outcomes resultant from educational methods.

**Conclusion:**

Video-based surgical education is effective in learning surgical skills within different levels of surgical training; however, superior study quality and follow-up is required to determine which aspects of video-based interventions are most impactful.

## Background

To date, the current model by which surgeons are trained relies heavily on apprenticeship-based Halstedian frameworks,^1^ through observation trainee surgeons acquire the knowledge, technical skills and critical judgement to successfully operate on patients. Although surgical expertise is integral to healthcare, challenges exist in the training of competent surgeons such as the implementation of work time directives,^2^ reduced opportunities for performing core procedures^3,4^ and budget cuts in healthcare organisations limiting investments into surgical training.^5^

With the advent of new video recording technologies, and improved computer systems, opportunity stands for innovation in surgical education. The use of educational videos offers many significant advantages including remote learning on demand, cost effectiveness, saved time and transport fees for healthcare professionals and students. Furthermore, with the development of validated checklists in the assessment of surgical skills, the opportunity lies for the extension of video-based learning to assessment and further.^6^ The utility of videos in learning surgical skills became increasingly apparent during the COVID-19 pandemic.^7^

The aim of this study is to compare the effectiveness of video-based teaching interventions to alternative teaching methods, such as text and live workshop demonstrations. Moreover, it aims to evaluate the manner in which implementation of video-based teaching interventions is most effective in the acquisition of surgical skills by healthcare professionals.

## Methods

This review was conducted according to PRISMA (Preferred Reporting Items for Systematic Reviews and Meta-analyses) guidelines^8^ and registered on “PROSPERO” the international prospective register of systematic reviews prior to the study commencement (Registration ID: CRD42021233836).

### Eligibility

Inclusion for research analysed in this systematic review was limited to primary research. All studies with original data relating to surgical skill acquisition through the utilisation of video technology were included. Inclusion criteria for study participants were medical students, surgical trainees, surgeons of all grades and specialities, healthcare students and healthcare professionals. The control group was the cohort receiving alternative methods of training in the acquisition of surgical skills. Papers were included unrestricted by publication date however study inclusion was limited to those which could be obtained in the English language if the mentioned criteria were met. Studies that did not utilise video in the direct demonstration and teaching of a surgical skill or procedural steps were not included. Studies regarding the improvement of surgical skill following video-based feedback, patient education or live streaming the surgical teaching were not included, as this review is assessing the use of video in the initial learning and acquisition of surgical skill in healthcare professionals/students.

### Search Methods

3 Databases were searched for this systematic review: MEDLINE (Via PubMed), Embase (via OVID) and Cochrane libraries, Clinicaltrials.gov was also searched for eligible ongoing studies. The search was conducted using the terms: Video* AND surgical AND teaching OR skill OR education. The listed databases were searched from inception date to 28/02/21. Effort was undertaken in analysing reference lists from articles meeting the inclusion criteria to identify additional eligible studies.

### Study Selection

Study selection was performed by two independent authors and with a third more senior reviewer to settle disagreements during study selection. Three levels of review were completed, firstly title screening and abstract screening to eliminate ineligible articles and lastly a full analysis of the manuscript, disagreements following discussion regarding eligibility were settled by the third reviewer. Articles which met the eligibility criteria also had their reference lists screened for additional eligible articles. Table 1

**Table 1.** Summary of included studies.

Study reference	Study Type	Participant information	Type of Intervention	Method of Assessment	Duration of Education	Outcomes	Validity	Level of Effectiveness	MERSQI score
*N.Akl et al.* ^15^	Prospective observational study	N = 12 Obstetrics and gynaecology residents (11), Practicing Obstetrician and gynaecologist (1)	Laparoscopic suturing Pre/ post intervention assessment Dry lab instructional video Singe study cohort, all given video intervention	Time taken to perform tasks/ number of movements	One session - 6-minute instructional video, 25 minutes to complete 5 tasks	Post video education median time to perform all timed tasks improved by 20% (115.5 seconds, p 5 .009) 6 movements to 4 movements	Content = N Response Processes = N Internal structure = N Relations to other variables = N Consequences = N	LOE = 2	9.5
*Chien et al.* ^16^	Randomised controlled trial	N = 40 First year medical students	Laceration repair – suturing skills Post intervention assessment Echo 360 Lecture capture software (overhead camera) Live workshop group vs. Video based learning group	Combination of various assessment criterion, 22-point suture task checklist	2 hours of training – 20-minute video and 1 hour and 40 minutes for practice	No significant difference in suturing scores at one and three months between Video based learning groups (*P* =.549) and live workshop learning (*P* =.8979) in student laceration repair performance	Content = 2 Response Processes = 1 Internal structure = 1 Relations to other variables = N Consequences = N	LOE = N/A	9.5
*Dantas et al.* ^17^	Randomised controlled trial	N = 30 30 first year dentistry students	Dental surgery Post intervention Assessment and evaluation trials testing retention Active learning (interactive meeting) (1) vs. Text (2) vs. Text with video (3) Video group supplemented with reading material	17 item checklists, each test scored out of 10 Assessed by one of four blinded calibrated observers	15-minute video of surgical procedure – 24-hour time period to study	Active learning group significantly better outcomes immediately (*P*<.05), All results similar after 60 days of first practice	Content = N Response Processes = 1 Internal structure= N Relations to other variables = 0 Consequences = N	LOE = N/A	10.5
*Flores et al.* ^18^	Randomised controlled trial	N = 32 Medical students (Preclinical)	Plastic surgery techniques Pre/post intervention analysis with crossover Digital animation vs. Textbook	Specific Validated surgery competency scale - graded in a blinded manner by two craniofacial surgeons Student evaluation of educational quality survey	20 minutes to review educational material	Pre intervention scores did not differ significantly between the participants (*P* = .74), Post intervention demonstrated significantly higher performance in the animation group compared to the text group (*P *< .001) Significantly higher scores in animation group across all metrics	Content = N Response Processes = 2 Internal structure= N Relations to other variables= 0 Consequences = N	LOE = 2	13.0
*Hamour et al.* ^19^	Longitudinal Cohort study	N = 6 Head and neck surgery residents	Thyroid lobectomy Pre/post intervention analysis Video teaching module – 15 minutes including instructional text	Assessment sheet, Observational Clinical Human Reliability Assessment (OCHRA system) Completed by the same observing head and neck surgeon in a blinded fashion	15-minute video-based teaching module after 3 weeks	Statistically significant reduction (49%) in errors post intervention (*P* < .05) 52% less staff takeover events (*P *< .05)	Content = 2 Response Processes = N Internal structure = N Relations to other variables = 0 Consequences = N	LOE = 2	12.2
*Lwin et al.* ^20^	Randomised controlled trial	N = 50 House surgeons	Basic surgical skills Pre/Post intervention analysis Interactive video-based instruction (IVBI) – Voice over narration and on-screen annotation vs. instructor led teaching sessions	Objective Structured Assessment of Technical Skills (OSATS) – Used pre and post intervention Exit survey for satisfaction scores in IVBI group.	1 hour teaching session – 8 to 1 instructor to participants in non IVBI group	Self-directed IVBI was shown to be as effective as instructor-led teaching upon pre-defined non inferiority margin (.72 points below, 90% confidence interval: 3.8 to 5.2) All participants selected for the IVBI group “some- what agree” or “strongly agree” that IVBI should be included in the House Surgeon curriculum	Content = 2 Response Processes = N Internal structure = N Relations to other variables = 1 Consequences = N	LOE = 2	12.0
*Mishra et al.* ^21^	Randomised controlled trial	N = 16 Ophthalmology residents	Ophthalmologic procedures: Blepharoplasty and laceration repair Post intervention analysis Video of attending surgeon performing procedure on pic eyelid, fully narrated Video and text (Group 1) group vs Text instructions alone (Group 2)	Time to completion and aesthetic grade (1-5 given by blinded attending Physician) Participant satisfaction survey	5 days before wet laboratory	Better aesthetic grades in video group for laceration repair (*P*=.038), however not for blepharoplasty (*P *= .492) 100% of residents who completed the survey said they would like videos prior to wet laboratory and Oculoplastic procedures	Content = N Response Processes = N Internal structure = N Relations to other variables= 1 Consequences = N	LOE = 1	10.4
*Patel et al.* ^22^	Randomised controlled trial	N = 27 Obstetrics and Gynaecology residents	Laceration repair Pre/post intervention written test/ post intervention practical test Instructional video supplemented with textbook chapter vs. live teaching session	Written examination – practical exam scored using a procedure checklist Examiners were not blinded	4 weeks between intervention and testing	No significant difference between live teaching group and video group – no pre intervention scores for procedure Significant improvement pre/post intervention written scores in Post graduate year 1 residents (P < .001)	Content = N Response Processes = N Internal structure= 1 Relations to other variables= 1 Consequences = N	LOE = N/A	10.0
*Lohre et al.* ^23^	Randomised controlled trial	N = 18 Senior orthopaedic surgery residents	Reverse shoulder arthroplasty Pre post intervention knowledge assessment/ Post intervention surgical skills assessment Immersive virtual reality (IVR) group vs. Video learning (VL) group	Objective Structured Assessment of Technical Skills (OSATS) tool and Global rating scale (GRS) Participant learning assessment questionnaires Three expert surgeons acted as evaluators	Not clearly defined	IVR group significantly higher mean (SD) cumulative OSATS scores than the VL group (*P* < .001) GRS total and individual domain scores were not significantly different between groups	Content = 2 Response Processes = 0 Internal structure = 2 Relations to other variables =1 Consequences = N	LOE = 2	14.2
*Norris et al.* ^24^	Randomised controlled trial	N = 24 Junior Gynaecology residents	Assessment of laparoscopic Salpingo-oophorectomy (LSO) performance Post intervention assessment Surgical teaching video group vs. traditional residency training group	Objective Structured Assessment of Technical Skills (OSATS; 20 points) tool LSO specific tool (30 points) Secondary outcomes: Knowledge questionnaire scores and self-assessed confidence scores	1 Week prior to date of surgery	No significant differences in mean (standard deviation) OSATS scores (*P* = .30) or LSO-specific tool scores (*P* = .24) Significant difference in mean knowledge scores/ knowledge of pelvic anatomy between the video and the traditional training (*P* = .01, *P* = .04).	Content = 2 Response Processes = 0 Internal structure = 2 Relations to other variables = 0 Consequences = N	LOE = 1	12.5
*Yang et al.* ^25^	Randomised controlled trial	N = 60 50 surgeons and 10 nurses	Robotic surgical skills on DV-simulator, Match board 2 (MB) and Thread the Rings 1 (TR) Pre and post intervention assessment of robotic surgical skills. Controller of events on simulator and robot (CESIR) video-based learning group (Group A) vs Instructor demonstration (Group B)	dV-Trainer automatic scoring system (M scores)	Completed over a single session/ unclear	Group A scores significantly higher post intervention in both MB and TR (*P* < .05)	Content = 0 Response Processes = 0 Internal structure = 2 Relations to other variables = N Consequences = N	LOE = 2	14.2
*Crawshaw et al.* ^26^	Randomised controlled trial	N = 54 General surgery residents	laparoscopic colorectal resection – Post intervention analysis 18-minute narrated instructional Video group vs. independent study group	Validated Global assessment scale completed by attending surgeon	2 days	Residents in the video group scored significantly higher in total score (mean: 46.8 vs 42.3; *P* = .002) Less verbal support needed in video group	Content = 2 Response Processes = 0 Internal structure = 2 Relations to other variables = 2 Consequences = 0	LOE = 3	13.0
*Sakamoto et al.* ^27^	Randomised controlled trials	N = 86 Fifth year medical students	Microsurgery Post intervention analysis 10-minute instructional video vs. 20-minute suturing practice Video group (Group 1) vs. Simulation group (Group 2)	The Nagoya University Micro-Suturing Assessment System (NUMSAS) Assessed by two blinded expert neurosurgeons Time to complete task Neo-five-factor inventory (FFI) personality test	1 hour	No significant difference between video-learning groups and simulator – learning group (P = .11287) High extraversion scores associated with shorter time to complete suturing assessment	Content = N Response Processes = N Internal structure = N Relations to other variables= 1 Consequences= N	LOE = N/A	10.0
*Saun et al.* ^28^	Randomised controlled trial	N = 30 Fourth year medical students	Chest tube insertion Pre and post intervention analysis instructional video Video group vs. instructor/lecture group	Pre/post intervention questionnaire Adapted Objective structured clinical exam (OSCE) recorded and scored by research assistants	16 minutes (Video)/17 minutes (Didactic lecture)	No significant difference in procedural OSCE scores/questionnaire Pre- intervention. Significantly higher post-intervention questionnaire scores compared to the didactic group, demonstrating a greater acquisition of knowledge.	Content = N Response Processes = 0 Internal structure= 2 Relations to other variables = N Consequences = N	LOE = 2	11.5
*Shim et al.* ^29^	Randomised controlled trial	N = 45 Medical students	Vesicourethral anastomosis Post intervention analysis Robotic surgical skills DV-simulator Proctored video group (Group I) vs. Video group (Group II) vs. independent training group	Automated scoring algorithm – validated	60 minutes daily (How many days unclear), exercise repeated over 80 times	Significant difference between time taken to complete task and score >70 in video groups and independent groups (*P *< .001), no significant difference when comparing group I and group II, which suggests that the educational video may be as beneficial as expert proctoring (P = .291)	Content = 0 Response Processes = 0 Internal structure = 2 Relations to other variables = N Consequences = N	LOE = 2	12.0
*Summers et al.* ^30^	Randomised controlled trial	N = 69 First year medical students	Knot tying and suturing Pre and post intervention assessment Instructor led videotape review Didactic Instructor demonstration vs. videotape instruction, or vs. multimedia Computer based training instruction	Written multiple choice test, designed by National board of medical examiners Performance based skills assessment	4 hours and 30 minutes (Including: introduction, knot tying and suturing)	CBT groups demonstrated statistically significant (P < .01) enhancement of technical skills compared with the didactic group. After 1 month, a calculated performance quotient revealed statistically significant (*P* < .01) improvement only in the CBT group.	Content = N Response Processes = 0 Internal structure = 0 Relations to other variables = 2 Consequences = N	LOE = 2	10.5
*Takiguchi et al.* ^31^	Randomised controlled trial	N = 36 Medical students and 1st year trainee doctors	Endoscopic knot tying Pre/post intervention assessment Repeated viewing of video at various speeds Instructor training (IT) group (Group A) vs IT and Self training (Group B) vs IT and cyber visual training (Group C)	Virtual reality system, MIST-VR Time taken to complete	10 – 25 minutes depending on group allocation	Time required for knot tying was significantly shorter in group C than the time in group A (*P* = .0375) No significant difference between groups B and C There were no significant differences in the parameters assessed using the MIST-VR	Content = N Response Processes = N Internal structure = N Relations to other variables = 0 Consequences = N	LOE = 2	12.1
*Sun et al.* ^32^	Randomised controlled trial	N = 20 Novice dental students	Oral surgery technique Post intervention assessment 10-minute instructional video Video instruction group vs. text instruction group	Recording of procedure assessed according to The Arch Bar Placement Assessment Scale (ABPAS) scored from 0 -19 Student perceptions questionnaire	10 minutes video/text instruction, 45 minutes examination	Post instructional skill scores not significantly different (P = .46), Significant preference for video-based instruction (P < .05)	Content = 0 Response Processes = N Internal structure = 1 Relations to other variables = N Consequences = N	LOE = 1	10.0
*Xeroulis et al.* ^33^	Randomised controlled trial	N = 60 First year medical students	Suturing and knot tying skill Pre/Post intervention/retention assessment Computer based video instruction (CBVI) Control vs. Video based vs. concurrent feedback vs. Summary feedback	Global rating scale pre/post retention trials graded by 2 blinded experts (OSATS) Imperial college surgical assessment, measured number of movements and total time	1 hour of practice	Mean GRS scores between pre- test and post-test demonstrated a significant improvement in all groups (*P *< .001), Mean GRS scores between pre- test and retention-test were significant only for the CBVI and summary feedback groups (*P *= .037).	Content = 2 Response Processes = 0 Internal structure = 2 Relations to other variables=N Consequences = N	LOE = 2	11.5
*Yee et al.* ^34^	Randomised controlled trial	N = 22 Surgical residents	Open carpal tunnel release Pre/post study and intervention assessment Edited 9-minute recording of open carpal tunnel release procedure Intraoperative recording with voice over description of operative steps vs. Operative text group	OSATS graded by 3 blinded board-certified plastic surgeons Pre/post intervention questionnaires to track confidence in surgical skills	40 minutes	Surgical video group committed fewer operative errors (*P* = .043), and were more confident in their abilities post procedure (*P* = .043)	Content = 2 Response Processes = 0 Internal structure = 2 Relations to other variables =2 Consequences = N	LOE=2	14.5
*Yeung et al.* ^35^	Randomised controlled trial	N = 80 First and second year medical students	Laparoscopic square knot on box trainer Post intervention assessment Instructional video Text group vs. video group	Time for completion, numbers of attempts at task, numbers of people who expressed understanding of task	10 minutes to review materials 15-minute time limit on box trainer	Time for completion were not statistically different in the two groups (*P* = .38). However, time to review the material (407 s text v. 258 s video, *P* = .001), number of attempts at the task (15 text v. 5 video had n > 2 attempts, *P* = .01), Number who expressed understanding (35% text v. 59% video, *P* = .047) were significantly different	Content = N Response Processes = N Internal structure = N Relations to other variables = 0 Consequences = N	LOE = 2	10.0
*Yoganathan et al.* ^36^	Randomised controlled trial	N = 40 Foundation year one doctors	Single handed reef knot Post intervention assessment 360 Degree video technology 3D Virtual reality (VR) Video group vs 2D video group	Royal college of surgeons, Basic surgical skills course marking criteria assessed by blinded examiner	15 minutes for video, 5 minutes for practice No time limit with completion of the knot	Scores were significantly better in the VR Group (median knot score 5.0 vs 4.0 *P* = .04) 25% More of the VR group cohort were able to construct a completed reef knot	Content = N Response Processes = N Internal structure = 2 Relations to other variables = 0 Consequences = N	LOE = 2	13

### Data Extraction

The extraction of data from the eligible studies was completed and checked by both authors, disagreements following discussion were resolved by the third reviewer. Information extracted from the studies included: study type, type of educational video, participant information, duration of education, method of assessment and outcome data analysed for methodological validity and educational effectiveness using Messicks and McGhagie’s frameworks. For data extraction, a standard template was decided on prior to analysis of studies (Table 2).

**Table 2.** Template used for data extraction.

Study Type	Participant information	Type of Intervention	Method of Assessment	Duration of Education – Length of video	Outcomes
•E.g. Cohort study, Randomised/Non-Randomised controlled trial	•N = Number of participants •Participant background	•Procedure being practiced •Pre/Post intervention assessment •Type of video •The study groups	Criteria for assessment •Range of scores •Who was involved? •Method of standardisation	•Duration of education and assessment if specified in study	•Statistical significance

### Data Analysis

Eligible studies were analysed according to Messicks criterion (Table 3)^9,10^ testing for validity of the study assessments on five different facets: content, response processes, internal structure, relation to other variables and consequences. Each of these parameters were graded according to the Beckman et al. rating scale (Table 4). The level of validity in each domain ranged from N, where there was no discussion of the source of validity, to two whereby data strongly supported the source of validity.^11^ Validity scoring was performed by two independent authors, a senior author was present to reconcile any disagreements in scoring. Scores were subsequently reviewed by all co-authors prior to completion of this manuscript. The level of effectiveness (LoE) of the educational models in the respective studies were quantified using an adapted version of McGhagie’s translational outcomes, to serve video-based educational models as opposed to simulation based educational models. In the present review McGhagie’s initial translational outcomes have been extended to five outcomes (Table 5).^12,13^

**Table 3.** Messick’s modern concept of validity framework.

Parameter	Definition	Examples
Content	Test items are relevant and representative of the intended construct	Using expert opinions to ensure all domains are accurately represented
Response Processes	Thought processes and actions of subjects and observers are made in accordance with the intended construct	Quality control of assessments, such as in standardising test administration and minimising examiner bias
Internal Structure	Test scores across tasks can be reliably reproduced	Calculating inter-item reliability and test-retest reliability
Relations to Other Variables	Test scores correlate with external, independent measures which share a theoretical relationship	Comparing scores between groups with different levels of experience in the tested skill
Consequences	The impact of using the assessment	Determining the pass-fail score and considerations for the subject on obtaining a pass or fail

**Table 4.** Rating scale by Beckman et al.

Score	Definition
N	N = no discussion of source of validity evidence presented
0	0 = discussion of source of validity but no data
1	1 = data weakly supports source of validity or is limited
2	2 = data strongly supports source of validity

**Table 5.** McGhagie’s Translational outcomes of simulation-based mastery learning (adapted version).

Parameter	Definition	Examples	Rating
Internal acceptability	The trainee’s satisfaction with using the video-based learning platform	Favourable responses from feedback forms or post training survey questionnaires	Level 1
Contained effects	Changes in performance of surgical skills	Development of knowledge and/or skills as measured by a form of assessment	Level 2
Down-stream effects	Behavioural changes in the clinical context	Adopting safer patient-care practices	Level 3
Target effects	Direct changes to patient outcomes	Reduced rates of surgical complications	Level 4
Collateral effects	Changes on a wider, systemic level	Cost saving; skill retention	Level 5

### Risk of Bias

Study quality was assessed and quantified using the validated medical education research study quality instrument (MERSQI),^14^ the MERSQI tool consists of six scoring domains created to assess different aspects of medical education research. Domains include study design, sampling, type of data, validity of assessment instruments, data analysis, and study outcomes. The maximum score per study is contingent on the study purpose, hence for this systematic review scores were standardised over a common denominator of 18 enabling comparison. Due to the nature of the studies and the heterogeneity of the study designs and outcome measures, qualitative analysis of the articles was performed.

## Results

### Study Selection

**Figure 1.**
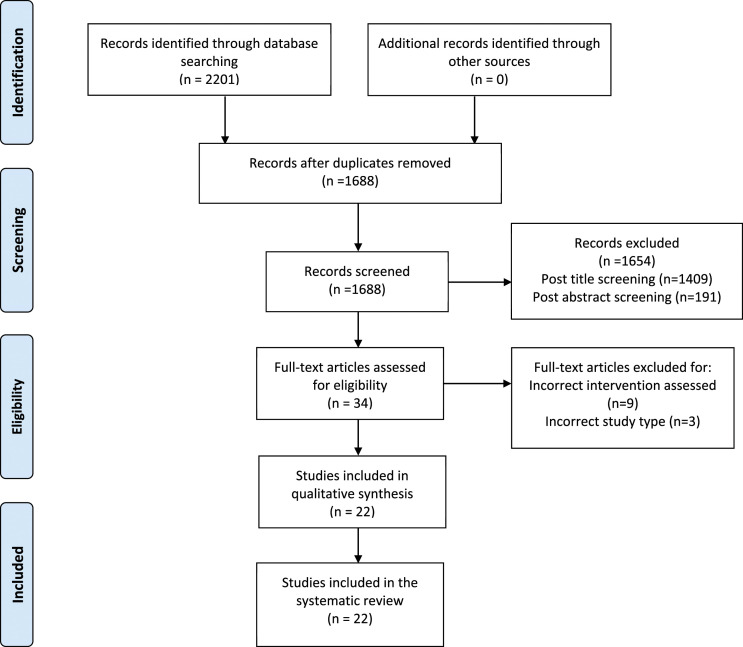
Study selection. Taken from: PRISMA flow sheet Moher D, Liberati A, Tetzlaff J, Altman DG, The PRISMA Group (2009). Preferred Reporting Items for Systematic Reviews and Meta-Analyses: The PRISMA Statement. PLoS Med 6(7): e1000097. doi:10.1371/journal. pmed1000097.

The search produced 2201 results, following duplicate removal 1688 studies remained for screening. The 1688 studies underwent title and abstract screening, 34 remaining studies underwent a full text screening, after a thorough analysis of the 34 articles, a further 12 articles were excluded, 22 articles remained for inclusion (Figure 1).

### Study Characteristics

Of the included studies 20/22 were randomised controlled trials and 2/22 were prospective observational studies. The number of intervention groups also differed between studies. The total number of participants across the included studies within this systematic review was 797.

### Types of Intervention

The surgical skill focus being learnt and tested varied between studies and can be broadly divided into three categories : full surgical procedures 4/22 (20%),^19,23,26,34^ general surgical skills (suturing, knot tying etc.) 11/22 (50%)^15,16,20,22,25,27,30,31,33,35,36^ and simulated surgical procedures 5/22 (22.7%).^17,18,21,23,28,29,32^ Studies also varied on method of assessment, some articles assessed skills pre intervention and post intervention 12/22 (54.5%), whereas others solely examined skills post intervention 10/22 (45.5%).

### Features of Video-Based Interventions

Multiple methods were used in the delivery of video-based interventions, the most common simply included 2-dimensional video with narrations describing procedural steps which 11/22 (50%) mentioned to have utilised either through audio or on-screen narration. Animation technology was implemented in 2/22 studies (9.1%),^18,24^ a further 2/22 (9.1%) also used still images throughout the procedural video to augment certain instructional steps.^31,32^

### Level of Effectiveness

Of the 22 papers analysed according to McGhagie’s translational outcomes,^13^ 3/22 (13.6%), studies scored an LOE of 1 indicating positive trainee satisfaction relating to the intervention, typically recorded via feedback forms.14/22 (63.6%) papers scored an LOE of 2 suggesting a statistically significant improvement in surgical skill as quantified by a form of examination, either practical or knowledge based. 1/22 (4.5%) of studies scored an LOE of 3, indicating an improvement of performance in the clinical context. Crawshaw et al.^26^ assessed the effectiveness of incorporating a comprehensive instructional video prior to the performance of a right laparoscopic colectomy, which improved overall surgical performance and operative independence within surgical residents. Lastly, 4/22 (18.2%) of the included studies scored N/A, this was allocated when no statistically significant positive outcome was reported relating to the administration of the educational intervention. None of the articles included in this study reported a LOE of 4 or 5 whereby patient outcomes were quantifiably affected or changes witnessed on a systemic level. This LOE requires an additional level of analysis, through widescale long term implementation of the educational interventions, which was not conducted in the included studies. Rather studies assessed educational interventions on a more immediate level, such as in trainee satisfaction and improvement of knowledge and skills.

The majority of studies within this systematic review assessed outcomes through validated practical examination checklists, completed by blinded expert examiners testing multiple aspects of good surgical practice such as proper motion handling, knowledge of instruments, and flow of operation.^37^ Subjective student experience surveys, designed to assess the allocated intervention on factors such as: student satisfaction, clarity of teaching materials and whether the participant deems the educational method effective^38^ were also incorporated in 7/22 studies. Of the 7 studies which incorporated student surveys, 6 (85.7%) demonstrated favourable responses towards the video-based teaching intervention.

### Video Based Interventions vs. Conventional Methods

Conventional teaching methods were used in 9/22 (40.9%) studies within this systematic review, defined in this context as: live workshops, textbook use and instructor led teaching sessions/lectures. In 7/9 (77.8%) of the studies comparing video-based interventions to conventional methods, a LOE of 1 or 2 was recorded, favouring video-based interventions. Overlying themes which made video-based interventions a superior modality, include the ability of the participant able to control replay speed, pause and advance to focus on certain aspects.^20,21^ Further benefits of the use of educational video were in the post recording edits, educational video can be enhanced through the use of narrated diagrams, physical demonstration and the ability to highlight specific aspects augmenting understanding among participants.^28^

### Video Based Learning vs. Virtual reality

Two of the included studies within this systematic review compared typical video-based education to virtual reality,^23,36^ Lohre et al.^23^ assessed orthopaedic residents in performing a reverse shoulder arthroplasty, results revealed that the immersive virtual reality had a significantly higher mean OSATS scores than the video group (15.9 [2.5] vs 9.4 [3.2]; difference, 6.9; 95% CI, 3.3-9.7; P < .001). Yoganathan et al.^36^ demonstrated similar findings, although the study focussed on acquisition of basic surgical skills as opposed to a complete procedure. Scores were significantly superior in the virtual reality (VR) group (median knot score 5.0 vs 4.0 *P* = .04) than the video-based intervention group, with 25% more of the cohort being able to construct a completed reef knot.

### MERSQI Scores

All the included studies above were graded according to the MERSQI tool which has a maximum possible score of 18, although for certain studies adjustments were made as not all assessment fields were applicable and a common denominator of 18 was implemented. The mean MERSQI score across all studies was 11.66 (64.7%) (range = 9.5 -14.5 (52.7% - 80.6%), Standard deviation = 1.55 (8.6%)).

### Validity of Evaluation Methods

Of the analysed studies within this systematic review, 14/22 (63.6%) studies reported with data strongly supporting the validity of the evaluation instrument (Table 4) on at least one of Messicks parameters (Table 3). 9/22 (40.9%) reported grade 2 validity on one parameter, 3/22 (13.6%) studies reported grade 2 validity on at least 2 parameters, and only 2/22 (9.1%) studies reported a grade 2 of validity on 3 domains. Validity was quantified through an analysis of the eligible papers and where specific information relating to the features of an evaluation instrument were not included, more information was obtained regarding the evaluation tool through the relevant paper obtained from reference reviews.

## Discussion

This systematic review has demonstrated that video-based teaching methods led to improved surgical skills/trainee satisfaction in 17/22 of the studies. Previous systematic reviews investigating the use of video in surgical education conducted by Green et al.^39^ and Ahmet et al.^40^ suggest a positive impact, however the eligibility criteria for both studies was limited to medical students and residents, conversely this study analysed research relating to any healthcare profession. The analysis of students from a range of different academic backgrounds, was considered more reflective of educational effectiveness of the video-based methods in surgical skill acquisition, due to the variability in the study cohort.

### Effective Implementation of Educational Video

An evident theme from this study was that video-based interventions which utilised virtual reality and simulation-based education proved more effective than standard videos. The immersive nature of virtual reality may render superior visuospatial awareness of technique, studies utilising virtual reality training had outcomes significantly superior to basic teaching video.^23,36^ Sakamoto et al. comparing simulation-based teaching interventions for microsurgical skills found video-based interventions to be inferior, improvement in the simulation group may have been due to the opportunity for hands on rehearsal.^27^

### Video Based Learning vs. Conventional Methods

Several features of video were markedly advantageous to conventional methods, themes identified upon analysis included: the ability to control replay speed and to navigate through videos directly to specific parts of focus,^20,21^ the highlighting of different anatomical structures, the imposition of schematic overlays and the use of audio/text narrations.^28^ Furthermore, through video editing attention can be directed towards the most important aspects of the teaching via auditory and visual annotations, enabling learners to achieve a superior understanding of taught concepts. Video editing also enables the removal of non-essential information reducing extraneous load, which by the cognitive load theory, would increase learning potential.^41,42^

### Advantages of Using Videos

Video-based teaching enables standardisation of educational materials, enabling not only a consistent foundation for future clinical trials but also consistently high-quality educational content for healthcare students/professionals. An example is the New England Journal of Medicine (NEJM) video series for learning surgical procedures. Videos are designed by multiple experts, they then undergo peer review prior to dissemination. Through the use of this video series, Saun et al. proved the superiority of standardised video-based teaching methods over didactic teaching for chest tube insertion, authors speculating the basis being due to the possible variability in the teaching approaches by different instructors, being overcome by standardised video.^28^ With advances in computer technology, trainees have access to high quality digital content at the click of a finger, creating opportunity for improvement of access to surgical education. Traditional learning of surgical techniques relies heavily on workshop-based interventions and the apprenticeship model,^43^ however research suggests significant advantages of augmenting surgical skills training with educational video through the ability to widely disseminate surgical video via the internet, study remotely and control aspects of material review.^39^ Video is also documented to enhance retention in the learner to a greater degree, a person typically retains 10-15% of that which is read, 10-20% of what is listened to, and 20-30% of what is viewed, however when audio and video materials are used in conjunction the retention of knowledge increases to 40-50%.^44^

### Disadvantages of Educational Videos

Although the employment of video-based teaching methods poses significant advantages, surgical video to date may not be able to replace traditional interactive teaching methods. Interactive teaching sessions have the additional element of live feedback and the ability to ask questions which solely relying on a teaching video would not provide.^28^ Further studies, assessing video-based interventions with additional features such as expert feedback during practicing of surgical skills and questions and answers, may enable a more comprehensive comparison of teaching methods.

### Recommendations

Most studies included in this systematic review (17/22) demonstrated improved examination scores and positive trainee perceptions resulting from video-based training. However, whether video-based education had advantages which translated to improved performance in the clinical context was undocumented, except in a study by Crawshaw et al.^26^ The extension of studies to assess surgical performance beyond a simulated environment can support the investment into video-based educational resources and curricula for trainees. Conduction of such research could be in retrospective analysis of complications and operative time prior to video-based education and after. Alternatively, patient outcomes in a surgical setting could be investigated through a randomised controlled trial where video-based education is employed.

In addressing low validity scores, the design of checklists and study protocols (with validity criterion accounted for) would have improved external validity of included studies. The advantages of the sources of validity mentioned are evident, an example of this is in comparing a study by Chien et al.^16^ which scored high on content validity, with Dantas et al.^17^ which showed no evidence of content validity. Chien et al.^16^ used a scoring checklist with domains based upon predefined guidelines in published literature accounting for expert opinion, whereas Dantas et al.^17^ did not clearly define where test items were derived.

The establishment of a study protocol checklist, which accounts for validity and investigation of similar outcomes, would increase study quality and homogeneity allowing a more rigorous evaluation of video-based surgical education. The literature reports the existence of standardised educational video libraries, for example the NEJM video series. The uniformity in composition video features, if applied across multiple study groups, would further prove the utility of video-based education.

### Limitations

Numerous recurring weaknesses were identified within the studies, firstly the low study quality as assessed by the MERSQI tool with the average quality quoted at 64.7%, Secondly, the use of validated evaluation instruments was lacking, none of the 22 studies reported validity on all five sources. Thirdly, none of the studies in this systematic review achieved higher than an LOE of 3, suggesting the lack of long term follow up. It may beneficial if future clinical trials could attempt to assess translational outcomes in terms of clinical practice. Also, multiple studies failed to specify the type of video used, as well as adjunctive features which may have been causative in the witnessed change/lack of change of assessment scores, thereby preventing a holistic analysis of video features which may have contributed to outcomes.

Due to the heterogeneity in study methodology of included papers, quantitative assessment of effectiveness of video-based interventions was not feasible and qualitative analysis of studies was performed. Studies differed drastically on multiple measures such as outcomes assessed limiting comparability. This study limited inclusion to papers which were in the English language.

## Conclusion

Video based interventions proved to have a positive effect in (17/22) 77.3% of the studies analysed in this systematic review. With the advent of new video technologies, and the ease of accessibility of video-based teaching, based on the findings of this review, the creation of standardised video programmes for the teaching of surgical skills is likely to enhance current teaching methods. It is evident from this study that video-based education overcomes many limitations in acquiring procedural knowledge and was favoured by trial participants. Although video may not be able to replace the interactive aspect of learning surgical skills, an analysis of the literature proved favourable outcomes in practical and knowledge examinations. Future research in the field should ensure evaluation tools are validated, ensuring reliability of any results, and aim to assess outcomes on a wider scale outside of the immediate study setting.
